# Advances in the Biofabrication of 3D Skin *in vitro*: Healthy and Pathological Models

**DOI:** 10.3389/fbioe.2018.00154

**Published:** 2018-10-31

**Authors:** Matthew J. Randall, Astrid Jüngel, Markus Rimann, Karin Wuertz-Kozak

**Affiliations:** ^1^Department of Health Science and Technology, Institute for Biomechanics, ETH Zürich, Zurich, Switzerland; ^2^Center of Experimental Rheumatology, University Clinic of Rheumatology, Balgrist University Hospital, University Hospital Zurich, Zurich, Switzerland; ^3^Competence Center TEDD, Institute of Chemistry and Biotechnology, Zurich University of Applied Sciences, Waedenswil, Switzerland; ^4^Center for Cell Biology & Tissue Engineering, Institute of Chemistry and Biotechnology, Zurich University of Applied Sciences, Waedenswil, Switzerland; ^5^Schön Clinic Munich Harlaching, Spine Center, Academic Teaching Hospital and Spine Research Institute of the Paracelsus Medical University Salzburg (AU), Munich, Germany; ^6^Department of Health Sciences, University of Potsdam, Potsdam, Germany

**Keywords:** 3D tissue model, skin, *in vitro*, bioprinting, electrospinning, skin disease, biofabrication, preclinical testing

## Abstract

The relevance for *in vitro* three-dimensional (3D) tissue culture of skin has been present for almost a century. From using skin biopsies in organ culture, to vascularized organotypic full-thickness reconstructed human skin equivalents, *in vitro* tissue regeneration of 3D skin has reached a golden era. However, the reconstruction of 3D skin still has room to grow and develop. The need for reproducible methodology, physiological structures and tissue architecture, and perfusable vasculature are only recently becoming a reality, though the addition of more complex structures such as glands and tactile corpuscles require advanced technologies. In this review, we will discuss the current methodology for biofabrication of 3D skin models and highlight the advantages and disadvantages of the existing systems as well as emphasize how new techniques can aid in the production of a truly physiologically relevant skin construct for preclinical innovation.

## Introduction

### Skin structure and function

**Figure 1:**
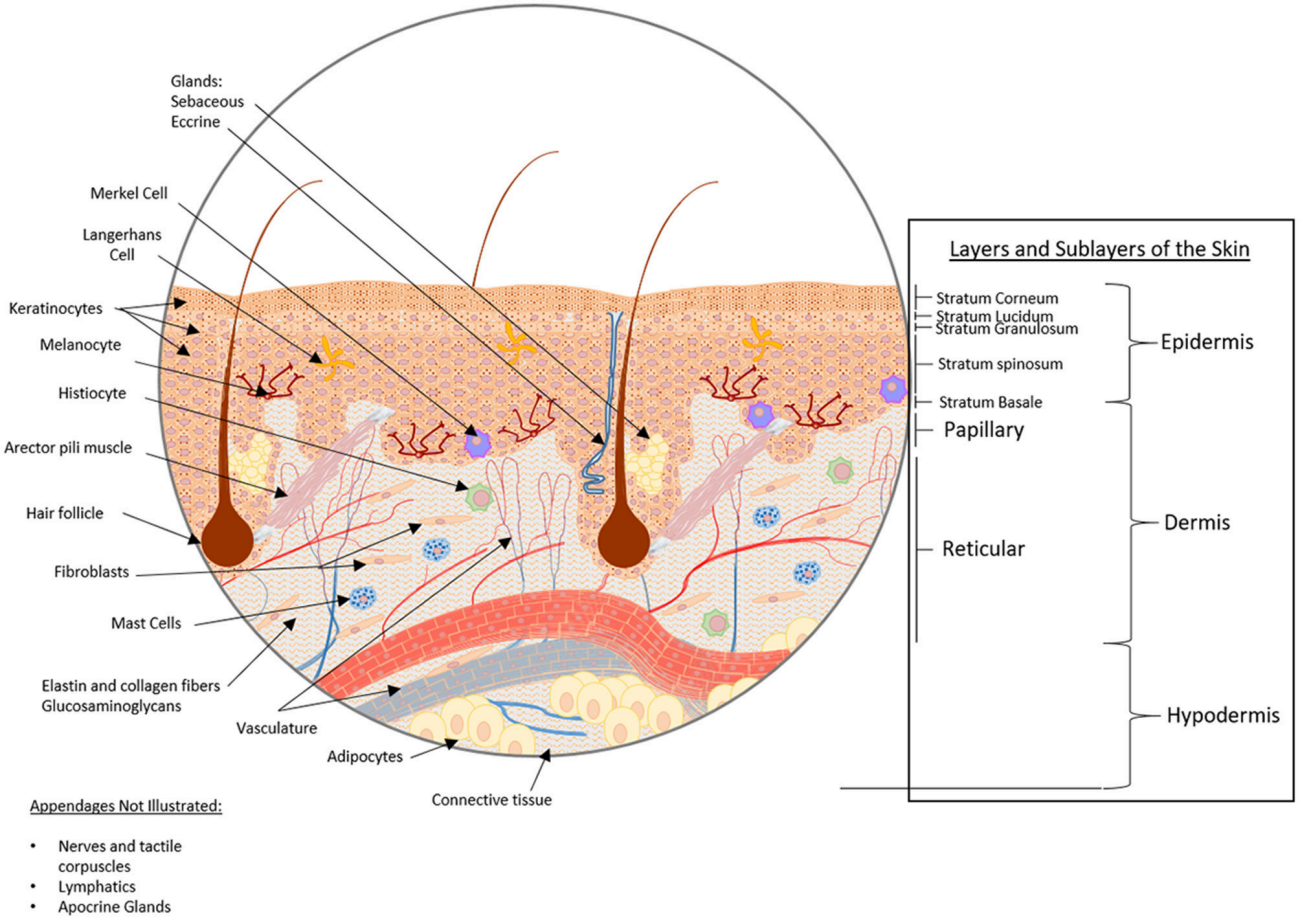
Healthy skin structure: layers, sublayers, and appendages/macrostructures. The skin consists of three main strata, from bottom to top: hypodermis, dermis, and epidermis. The hypodermis, also known as the subcutaneous tissue, is comprised of adipocytes within a mesh of connective tissue through which nerves and blood vessels traverse. Above the subcutaneous tissue is the dermis, a thick fibrous layer partitioned into two sub-layers, the papillary dermis and the reticular dermis. The dermis is primarily comprised of a fibrous scaffold within which fibroblasts, dermal dendrocytes, mast cells, and histiocytes can be found. Additionally, blood vessels, lymphatic networks, glands, hair follicles, and tactile corpuscles reside here. Above the dermis, the outermost stratum is the epidermis. The epidermis consists of five substrata: the stratum basale, stratum spinosum, stratum granulosum, stratum lucidium, and stratum corneum from deepest to most superficial. The cellular constituents of the epidermis are predominantly keratinocytes, though Langerhans cells, Merkel cells, and melanocytes can also be found in this layer.

The body's primary barrier against many environmental exposures, including organisms and chemicals, the skin, is the largest organ of the human body covering an area of ~2m^2^. The skin consists of three layers, from inner to outermost: hypodermis, dermis, and epidermis (Figure 1), each of which has a unique structure, composition and synergistic function. The hypodermis, also known as the subcutaneous tissue (adipocytes, nerves and blood vessels), functions as an insulator, shock absorber, and nutrient reservoir. The dermis, the thick fibrous layer, is partitioned into two sub-layers, the papillary and reticular dermis, although there is no sharp delineation between these layers. These layers consist of fibers (elastin and collagen) and glycosaminoglycans (GAGs), as well as many cell types (fibroblasts, dermal dendrocytes, mast cells, and histiocytes). Macrostructures found in this layer include blood vessels, lymphatics, and appendages (sebaceous, apocrine and eccrine glands, hair follicles and arector pili muscle, nerves and tactile corpuscles, and nails). The dermis functions as an insulator, mechano- and thermosensor, immunologic defense, as well as maintains proper hydration. Lastly, the outermost layer of the skin, the epidermis, consists of five stratified sublayers (from inner to outermost: stratum basale, stratum spinosum, stratum granulosum, stratum lucidum, and stratum corneum) consisting predominantly of keratinocytes, whereas Merkel cells, Langerhans cells, and melanocytes combined contribute to 5% of the cell population (Zaidi and Lanigan, 2010). The epidermis functions as a physical barrier, immune defense, and mechanosensor. All too often, the complexity of skin is simplified for *in vitro* experimentation, yet the complete structure and function of the skin as an organ is dependent on all layers, cells, and appendages, for proper function.

### From 2D to 3D models: the rationale

Current *in vitro* pathophysiological studies are conducted primarily in two-dimensional (2D) cell culture in which cells are grown as a monolayer on solid flat surfaces such as polystyrene or glass. This 2D system relies on a fluid medium that supplies the essential nutrients (amino acids, carbohydrates, vitamins, minerals), growth factors, hormones, gases (O_2_ and CO_2_) and that regulates the physical-chemical environment (pH, osmotic pressure, temperature) (Antoni et al., 2015). Although these 2D cell culture systems have played a key role in furthering our understanding of molecular signaling, cellular morphology, and drug discovery, not all results and conclusions from these 2D cell culture systems are translatable to physiological *in vivo* systems (Duval et al., 2017; Langhans, 2018). Additionally, 2D monolayers specifically lack the environmental factors (e.g. mechanical forces, spatial orientation, as well as physiological oxygen, nutrient and signaling gradients) associated within the three-dimensional (3D) *in vivo* environment. Moreover, cell-to-cell, cell-to-matrix, and cell-to-environment interfaces are important for physiologically relevant cell functions and the absence of these critical factors, such as that in 2D cell culture, impact cellular responses from morphology, proliferation, migration, and differentiation, to biochemical signaling as well as gene and protein expression (Ali et al., 2015; Antoni et al., 2015).

The abovementioned limitations of 2D cell culture models as unreliable predictors of *in vivo* drug efficacy and toxicity are further supported by the relative high drug failure rate during preclinical testing for diseases such as cancer (Hutchinson and Kirk, 2011; Boghaert et al., 2017). As a result, physiological 3D skin models are required to provide a better platform for predicting clinical outcomes for drug testing. One approach, “organ culture,” is the culturing of 3D human skin biopsies *ex vivo*. Although organ culture offers the ideal physiological construct, the practicality of its use is substandard due to limited tissue availability (specifically diseased tissue biopsies), donor-donor variability, and ethical considerations.

### A brief history of *in vitro* 3D skin models

In the middle of the twentieth century, just prior to the standardization of nutrient media, the culturing of 3D human skin biopsies *ex vivo* began (Medawar, 1948). Soon after, in the 1950's and −60's, the development of mixed- and single-type primary 2D cell cultures, including that of human keratinocytes (Wheeler et al., 1957), would prevail as standard lab practice over organ culture and 3D cell culture. Although 2D culture was simple and reproducible (Wheeler et al., 1957), the relevance for 3D constructs was evident (Ehrmann and Gey, 1956), and in the late 1970's a “living skin equivalent” implementing a dermis-like collagen hydrogel was described (Bell et al., 1979, 1981a,b). Further technical advances in the 80's and 90's, specifically electrospinning, would lead to the use of scaffolds for 3D cell culture (Li et al., 2002). Moreover, at present in the twenty-first century, with the implementation of 3D printing for use in biological applications (Lee et al., 2009), the simple hand-poured hydrogel matrix will become a relic, setting a new standard for the production of 3D tissue constructs.

This review aims to (1) describe the current methodology of 3D skin fabrication to date as well as the advantages, and limitations of the current techniques, and (2) to emphasize how advanced biofabrication techniques can drive the progression toward developing the next stage of reproducible and complex, physiologically relevant, 3D skin constructs for personalized medicine.

## Physiological skin model in 3D

In the modern age, it is crucial to develop 3D skin models. This notion is supported by an expanse of literature that demonstrates that cells respond more physiologically in 3D culture as compared to 2D (Hoarau-Vechot et al., 2018). Specifically, the biochemical signals, mechanical and structural properties of 3D constructs nearly resemble *in vivo* physiology. Moreover, primary skin-derived cells cultured in 3D systems not only mimic the *in vivo* environment, but also allow for personalized mechanistic and translational studies. Currently, two main types of engineered 3D tissue models exist: (1) Scaffold-free and (2) Scaffold-based.

### Scaffold-free 3D models

Scaffold-free models of 3D tissues, spheroids, are defined as non-adherent cell aggregates produced by the self-assembly of one or more cell types. Spheroids, also called microtissues, self-assemble by gravity force from monodispersed cells and replicate many features of organ and tumor tissues including deposition of extracellular matrix (ECM) proteins, cell-cell interactions, as well as the formation of nutrient, waste and gas (O_2_ and CO_2_) gradients (Hirschhaeuser et al., 2010). Using a scaffold-free 3D skin model is advantageous, due to low costs and reproducibility; such models are suitable for high throughput cellular function, cytotoxicity, or biochemical analyses. However, to obtain proper tissue architecture, the positioning of the cells dictates the production of a full thickness stratified epidermis. The production of spherical skin microtissues with different keratinocyte layers and a dermal fibroblasts core producing the ECM was described recently (Stroebel et al., 2016). Still, this model mimics skin tissue on a micro-scale and is fully immersed in media, which does not recapitulate the air-liquid-interface of physiological skin and in turn may affect compound penetration and action. Thus, an ideal skin equivalent should function as a physical barrier at the interface with a gaseous environment. The skin barrier is strongly dependent on the humidity and culture conditions in which it is created (Asbill et al., 2000). Though scaffold-free approaches for the production of micro-tissues are high throughput-compatible, the main application lies in anti-cancer drug development and toxicology assessment (Messner et al., 2013; Sant and Johnston, 2017). However, spheroids have been used to study skin cancer by the integration of melanoma cancer spheroids placed into skin equivalents (Vorsmann et al., 2013). In this respect, bioprinting offers the possibility to standardize the deposition of micro-tissues by the exact positioning of spheroids in the raised skin models (Jakab et al., 2010; Moldovan et al., 2017).

### Scaffold-based 3D models: natural or synthetic polymers and polymer combinations

Scaffold models of 3D tissues, when defined broadly, are cells grown in the presence of support scaffolds, either hydrogel-based support, or polymeric fiber-based support. These scaffolds can be comprised of natural, synthetic, or combinations of different polymers, and unlike scaffold-free models, represent a 3D construct that is structurally, mechanically and functionally similar to the biological tissue (Debels et al., 2015; Caddeo et al., 2017).

**Natural polymers** such as collagen, fibronectin, elastin, fibrin, silk, alginate, chitosan, fibrin, or GAGs (Mason et al., 2013; Lohmann et al., 2017), are typically non-cytotoxic and seldom illicit an inflammatory response, hence making them prime candidates for cell-laden *in vitro* 3D skin models. Collagen type I, as the primary component of dermal extracellular matrix (ECM), is the most commonly used natural polymer constituent in bioengineered skin. Specifically, the ability to extract collagen from natural sources makes it an attractive and physiologically relevant matrix candidate. Unfortunately, collagen and other biopolymers typically have weak mechanical properties due to the very high water content. For instance, collagen I is susceptible to physical contraction when integrated fibroblasts exert forces on the matrix (Moulin et al., 1996). This contraction is diminished by the removal of water from the scaffold by physical compression (Vidal et al., 2018). Furthermore, these hydrogels are prone to enzymatic degradation, e.g. by collagenases (MMP2, MMP9) and further by gelatinases, making them unstable for long-term cell culture (Karsdal et al., 2017). Additionally, some of the degradation products can induce chemotaxis of human fibroblasts (Chattopadhyay and Raines, 2014). Nevertheless, collagen-based bioengineered skin tissues are still the most commonly employed models to mimic both healthy and diseased skin *in vitro*. For that reason, additional techniques are implemented to improve the mechanical strength of natural scaffolds such as cross-linking. Common cross-linking techniques include either chemical cross-linking, using reactive components such as glutaraldehyde, or the amalgamation of natural products such as GAGs or fibrin (Brougham et al., 2015). An additional method utilized to stiffen naturally-derived collagen hydrogels is non-enzymatic glycation, whereby proteins are cross-linked by reducing sugars (such as glucose) during several chemical modifications (Mason et al., 2013). The disadvantage of this method is that the excess of sugar produces a cytotoxic hyperosmotic environment preventing the incorporation of cells during gel formation. Therefore, another tactic to enhance the biological and mechanical properties of scaffold models is the combination of two or more natural polymers. A hydrogel produced from cross-linked silk and collagen was described as an ideal dermal biomaterial (Vidal et al., 2018). This combination allows for the preservation of cell-binding domains from collagen while benefitting from the stabilizing properties of silk, which proved to be more resistant to time-dependent degradation and contraction when compared to collagen hydrogels. Moreover, the use of advanced biofabrication techniques, such as electrospinning and bioprinting, may also diminish the problems associated with contraction and chemical cross-linking. In 2018, de Torre et al. described electrospinning of clickable elastin-like recombinamers that do not require addition of potentially cytotoxic cross-linkers, while allowing for the incorporation of different functionalities (i.e. RGD motifs) to support cell adhesion and proliferation (Gonzalez de Torre et al., 2018). As outlined in this section many different biopolymers were used and developed to generate 3D skin models; however, collagen I, with the abovementioned limitations, is still predominantly used (Sahana and Rekha, 2018).

Besides the potential for correcting problems associated with hydrogel contraction and chemical cross-linking, bioprinting also permits the direct integration of cells, bioactive molecules and scaffold material, mainly hydrogels, in a layer-by-layer mode (Mironov et al., 2006). Initially, the scaffold materials used to bioprint hydrogels, with the ability to support 3D cell growth, included the natural polymers collagen I, alginate, or Matrigel® (Malda et al., 2013), and thus the term “bioink” was coined. Currently, three main technologies for the production of bioprinted tissues are employed: (1) laser-assisted bioprinting (LaBP), (2) inkjet-based, and (3) micro-extrusion-based bioprinting (Malda et al., 2013; Murphy and Atala, 2014; Ng et al., 2016; Huang et al., 2017; He et al., 2018). Advancements in bioprinting led to the development of tailor-made bioinks that provide optimal printing properties while maintaining cell-compatibility (Gungor-Ozkerim et al., 2018). Furthermore, bioprinting offers the possibility to co-print biomaterials with different mechanical properties to increase the scaffold's stability (Shim et al., 2011). With respect to bioprinting skin constructs, mainly natural-derived polymers are used. In 2017, Pourchet and colleagues printed a mixture of gelatin, alginate, and fibrin to generate a full-thickness skin model with a characteristic stratified epidermis (Pourchet et al., 2017). Lee et al. printed collagen I together with human primary dermal fibroblasts and epidermal keratinocytes in layers using an inkjet-based approach and demonstrated high cell viability (Lee et al., 2009). While these constructs did not possess a stratified epidermis, this could be improved with a combined air-liquid interface (ALI) approach (Lee et al., 2014). In the following years, other materials, such as photo-crosslinkable gelatin-methacryloyl (GelMA), were implemented with primary dermal fibroblasts and epidermal keratinocytes in ALI culture, resulting in full-thickness skin models with an epidermal-like structure, however this model did not fully recapitulate a stratified epidermis (Rimann et al., 2016). In order to increase the mechanical stability of the bioink, GelMA was mixed with collagen I and the enzyme tyrosinase (Ty). With this approach, Shi et al. (2018) could demonstrate not only increased mechanical stability of the Ty-crosslinked construct, but also an improved skin regeneration process. Nevertheless, a full thickness skin model was not present in this study.

Due to the poor mechanical properties and high batch-to-batch variability observed with natural polymers, **synthetic polymers** were developed (Antoine et al., 2014) including polyesters such as poly(ε-caprolactone) (PCL), polylactic acid, polyglycolic acid, polylactic-co-glycolic acid (PLGA), polyhydroxybutyrate, and polyethers such as polyethylene glycol (PEG) and PEG co-polymers. The advantages of using synthetic polymers resides in their adjustable physical properties (e.g. porosity, biodegradation, and stiffness/elasticity) to suit specific applications. PCL, for example, was used for the production of tissue scaffolds by both classical electrospinning (Sharif et al., 2018) and melt-electrospinning (Farrugia et al., 2013), in which a high porosity and interconnectivity promote cell invasion and synthesis of both collagen type I and fibronectin. Thus, electrospinning has been advantageous for the cost-effective production of scaffolds with large surface areas and high porosities that permit the fabrication of nanofibrous 3D skin models containing a matrix that bears high similarity to the native ECM (Wang et al., 2013). However, since synthetic polymers typically have poor cell adhesive properties they are regularly used in combination with natural polymers.

To circumvent the adhesive issues with synthetic polymers, **natural-synthetic polymer combinations** are used. Recently, studies fabricating collagen hydrogels cross-linked with PEG have demonstrated diminished contraction of hydrogels while maintaining cell viability of embedded primary dermal fibroblasts (Brougham et al., 2015; Lotz et al., 2017). Unfortunately, the cytotoxicity of chemical-crosslinked synthetic products such as glutaraldehyde or PEG is debatable (Vedadghavami et al., 2017; Rahmani Del Bakhshayesh et al., 2018). On the other hand, natural-synthetic polymer combinations have been tested with electrospinning. For instance, silk fibroin, a material known for its biocompatibility, yet comparatively good mechanical properties (Lee et al., 2014; Sheikh et al., 2015), was recently combined with PCL resulting in composites that possess a surface topography and chemistry that promoted fibroblast-induced collagen deposition (Lee et al., 2016b). Despite the attempts to improve hydrogel properties with the development of synthetic polymers, natural polymers appear to be essential for physiological significance of 3D constructs.

### Appendages and macro-structures

In addition to the structural and mechanical components of 3D tissue constructs, the inclusion of appendages and macrostructures such as vasculature and glands (Figure 1), is important for modeling physiological functions. A classical approach is the use of sacrificial polymers (e.g. gelatin, agarose, pluronic F-127) that are printed into a bulk hydrogel for later removal from the main matrix [e.g., collagen I, photopolymerized poly-(ethylene glycol) diacrylate], followed by perfusion of the produced channels in a subtractive manufacturing approach (Lee et al., 2010; Bertassoni et al., 2014). Over the past 4 years, the expansion of biofabrication techniques has aided production of vessels including bioprinting (Kolesky et al., 2014; Bibb et al., 2016), subtractive manufacturing with sacrificial electrospun polymers (Lee et al., 2016a) as well as many other approaches which are summarized in a review by Frueh et al. (2017). In fact, 3D skin constructs produced with vascular (Marino et al., 2014) and lymphatic networks (Marino et al., 2014; Gibot et al., 2017) are possible. Moreover, a commercially available vascularized skin model, Skin-VaSc-TERM® (ATERA SAS, France) is also in production. Despite these advances, there are major limitations for vascularization in 3D constructs including vessel diameter, reproducibility, adaptability, and the development of suitable perfusion culture conditions. Further combining and evolving of today's biofabrication techniques such as melt-electrospin writing (MESW) and 3D bioprinting could provide a means of producing microvasculature at the capillary scale (1–2 μm) and with precise placement of vessels in a reproducible manner. This allows for the controlled fabrication of duplicates from which comparisons can be made with respect to *in vitro* therapeutic screening, as well as the assessment of wound dressings, an application currently lacking an *in vitro* screening process. Furthermore, the production of macro-structures such as glands (i.e. sweat glands and sebaceous glands), tactile corpusicles, and hair are the next step toward a truly physiological skin model (Takagi et al., 2016). Although the *in vitro* growth of sebocytes (Barrault et al., 2012), eccrine glands (Klaka et al., 2017; Poblet et al., 2018), and hair follicles (Lee et al., 2018) has been tested in 3D, the biofabrication of these glands in reproducible constructs would be far superior with bioprinting (Huang et al., 2016). Overall, the biofabrication of these macro-structures are limited (Liu et al., 2016) and require further advancement to produce a physiological 3D skin model.

### Outlook of physiological 3D skin models

Although optimization of hydrogel fabrication is evident, there is, as of yet, no ideal bioink to produce the physiologically relevant hydrogel to mimic the structural, mechanical, and biochemical properties of actual skin. Thus, additional focus on hydrogel composition is necessary. Exploiting biochemical mechanisms for physiological polymer synthesis to produce bioink materials with enhanced physiological relevance may be one approach to consider. Moreover, combining technologies such as MESW and 3D bioprinting is possible, which provides additional opportunities to mix synthetic and natural scaffolds that create a tissue environment necessary for cell viability while simultaneously providing structural support.

Besides optimization of hydrogel composition, the addition of multiple cell types is challenging. Classically, fibroblasts and keratinocytes represent *in vitro* skin, though in the current era 3D skin constructs should be comprised of more than two cell types. The incorporation of melanocytes is more recently becoming commonplace due to the need for pigmented skin constructs in the cosmetic industry (discussed in more detail in the following chapter). Inappropriately, adipocytes, neurons, Langerhans cells, etc., are not considered central to the progression of *in vitro* 3D skin models. The microenvironments necessary to support many of these cell types require further investigation. Cells that reside within a specialized appendage, such as sebocytes, require further optimization of biofabrication techniques with respect to resolution of cell or polymer positioning. The incorporation of these specialized cell types as well as the fine-tuning of their respective microenvironments may be feasible with a combination of bioprinting and MESW. Furthermore, obtaining such specialized cells is an added challenge. Primary keratinocytes and fibroblasts can be easily isolated from “healthy” or diseased biopsies using standardized protocols, whereas specialized cells (e.g. Merkel cells) are fewer in number and lack specific isolation protocols. Progenitor cells may be an alternative, though use of stem cells harbors additional drawbacks such as extensive differentiation protocols with little standardization and the potential for inadequate phenotypes.

**Figure 2:**
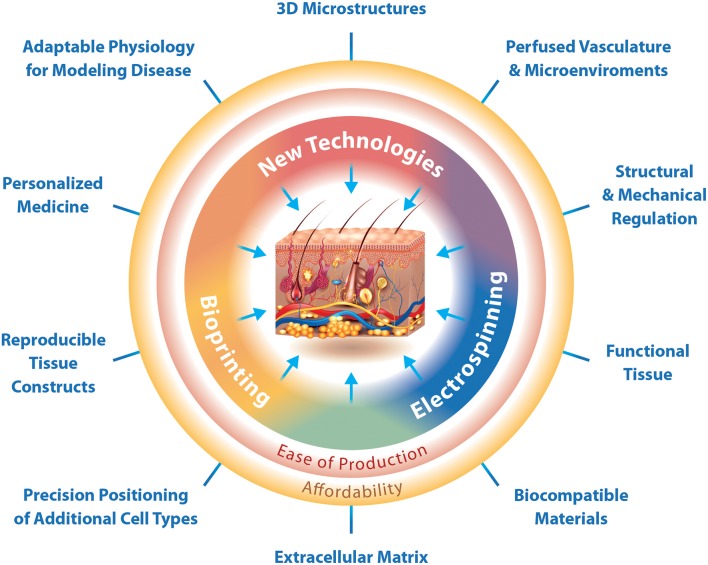
The interfaces at which biofabrication techniques enable the production of 3D physiological skin *in vitro*. Biofabrication techniques can function independently for a traditional purpose, but also in combination with other techniques to produce specialized structures. Bioprinting is specifically designed for the production of reproducible tissue constructs and precision positioning of cells. Electrospinning is designed for the production of functional tissues and use of biocompatible materials. New technologies should focus on the production of 3D microstructures. Where electrospinning and bioprinting meet we can create physiological extracellular matrix, while at the interface between electrospinning and new technologies is the ability to regulate tissue structure and mechanical properties as well as the production of vascular networks and adaptable microenvironments. The interface of bioprinting and new technologies would enable the production of 3D skin constructs with adaptable physiology for modeling disease and the possibility for preclinical testing of personalized medicines. Together the current technologies, bioprinting and electrospinning, as well as novel technologies can easily produce affordable 3D skin constructs that mimic physiology.

Moreover, following the optimization of the hydrogel and incorporation of all relevant cell types, the necessary appendages and macro-structures are still an added challenge. An appropriate vasculature with capillary structure to provide an *in vitro* environment that supplies oxygen and nutrients, and elimination of waste products in a physiological manner is necessary. Biofabrication techniques are critical for the development of these structures and while some techniques exist and facilitate the production of vascular skin models, there is yet to be a technique to produce vessels of capillary scale efficiently and with simultaneous cellularization and hydrogel fabrication. Specifically, MESW and 3D bioprinting have a great potential to produce adaptable physiological models with perfused vasculature and 3D macrostructures (Figure 2). In addition, defining an appropriate nutrient medium suitable for this method of tissue culture will be necessary to maintain the viability and differentiated state of cells for the duration of growth and experimentation. Despite the complex challenges, technologies are ever advancing and show great promise for the production of reproducible physiological 3D skin constructs with biocompatible and functional extracellular matrix, 3D microstructures, as well as perfused vasculature and lymphatics specifically for the *in vitro* identification and validation of personalized medicines (Figure 2).

## Skin disease models in 3D

### Use of 3D skin models for disease

Skin diseases are among the most prevalent and largest burdens of disease globally. The predominant types of skin diseases include fungal diseases, subcutaneous diseases, acne vulgaris, pruritus, eczema, impetigo, molluscum contagiosum/warts, and scabies (Hay et al., 2014). Currently, the primary means of identifying therapeutics for these diseases require the use of animal models (Avci et al., 2013), increasing not only the cost of drug development, but also the cost for consumers. In addition to the financial burden, ethical concerns encourage the development of *in vitro* 3D skin disease models such as the implementation of the 3R principles (Tornqvist et al., 2014). However, physiological interaction between keratinocytes, fibroblasts, immune cells, adipocytes and many other cell types are critical for the creation of physiological 3D skin constructs that can be easily adapted for disease modeling and personalized medicine.

### Current 3D skin disease models

Tissue damage, specific to the skin barrier, is the major source of infection and onset of disease. The skin can be damaged physically by blunt force, exposure to radiation, or by other means of dysfunction including but not limited to genetic and nutritional factors. The main types of disease models currently investigated include wound models, infection, cancer, and inflammation.

Presently, 3D skin models are used to both promote *in vivo* wound healing as a skin substitute (Curran and Plosker, 2002), and to identify crucial processes involved in typical wound healing (Egles et al., 2010). Chronic skin wounds, ulcers, demonstrate impaired healing and are typically associated with diabetes. In diabetic foot ulcers, increased Langerhans cells (Stojadinovic et al., 2013; Strom et al., 2014), dermal macrophages (Loots et al., 1998), and neutrophils (Vatankhah et al., 2017) positively correlate with disease severity in humans. Moreover, the importance for adipose tissue to promote closure of non-diabetic skin wounds was recently demonstrated in *drosophila* (Franz et al., 2018). Yet, currently the investigation of wound healing in 3D skin models is limited to wound closure by fibroblasts and keratinocytes. Thus, the incorporation of resident and circulating immune cell types as well as physiologically relevant delivery of nutrients are necessary to recapitulate diseases associated with chronic skin wounds. Furthermore, the growth of the resident dermal bacteria, *Staphylococcus aureus (S. aureus)* (Popov et al., 2014), as well as pathogenic bacteria, *Acinetobacter baumannii* (de Breij et al., 2012), have been established on uninjured 3D skin equivalents, and drug resistant strains of *S. aureus* have also been used in a wound infection model for therapeutic development (Ventress et al., 2016). The physiological relevance of 3D skin wound models (diabetic ulcers or chronic wounds) can progress with the addition of today's bioengineering techniques. Although, skin bioprinting is currently used to produce skin equivalents for *in vivo* wound treatment (summarized in a recent review; He et al., 2018), the technology has thus far not been applied to generate *in vitro* skin wound models. Still, it is clear that the bioprinting technology will have a substantial impact on development of *in vitro* skin wound models.

Prolonged and excessive sun exposure, and consequently, exposure to UV radiation (UVR) can irreversibly damage skin. This form of skin damage, photodamage, is a result of UVR-induced biochemical and structural changes in skin and manifests in a broad range of outcomes, from acute sunburn to chronic aging-related disease such as actinic keratosis, and further to cancers including melanoma and non-melanoma skin cancer. Animal models of photodamaged skin require high doses of UVR or prolonged exposures (Krishnasamy et al., 2017) that include strict ethical regulations and therefore the use of *in vitro* 3D skin models is gaining interest for these studies (Fernandez et al., 2014) (including that of commercially available skin equivalents such as MatTek EpiDerm™; Gruber et al., 2018). Skin cancers range from basal cell carcinoma to squamous cell carcinoma, as well as melanoma, and are one of the most common cancers for Caucasian people worldwide. Skin cancers are associated with photodamaged tissue and genetic mutations in proteins of the Sonic Hedgehog pathway (PTCH1, SMO), the mitogen-activated kinase pathway (RAS, RAF) and tumor suppressor gene p53 (Wei et al., 2015). These pathways are also associated with wound healing, due to regulation of proliferation and cell migration. Hill et al. recapitulated an invasive melanoma using rat melanoma cells in a 3D skin equivalent, thus demonstrating the current feasibility of introducing primary cells into a 3D skin model *in vitro* (Hill et al., 2015). Marconi et al. have demonstrated the importance of cell-cell and cell-ECM interactions in a 3D skin model of melanoma (Marconi et al., 2018). Moreover, modeling these cancers in reproducible and physiological 3D skin constructs would improve personalized drug screening. The use of patient-specific induced pluripotent stem cells (iPSCs) in the biofabrication of a 3D skin equivalent whereby each cell type is indicative of the patient has been demonstrated for normal skin tissue (Itoh et al., 2013; Gledhill et al., 2015) as well as for epidermolysis bullosa (Itoh et al., 2011). Alternatively, retroviral transfection can be used for reprogramming of somatic cells to pluripotent cells for the generation of diverse cell types though this might result in cell transformation and caution should be taken with respect to clinical use (Dixit et al., 2017). Stem cells have been bioprinted with high survivial rates to reproduce liver, cardiac and cartilage tissues after differentiation (Faulkner-Jones et al., 2015; Nguyen et al., 2017; Ong et al., 2017). The main challenge regarding bioprinting of stem cells is not the printing process itself, but rather providing the appropriate mechanical and biochemical cues that promote the specific differentiation and generation of the corresponding tissue-specific lineage.

All skin diseases from wounds to photodamage, and cancer to specific inflammatory diseases including psoriasis, dermatitis and scleroderma have characteristic inflammatory profiles. In an attempt to create a 3D skin equivalent that mimics inflammatory disease, researchers have integrated several immune cell types such as human CD4^+^ T cells (van den Bogaard et al., 2014), Jurkat T cells (Kuhbacher et al., 2017), Langerhans derived MUTZ-3 transformed cells (Ouwehand et al., 2012; Kosten et al., 2015), primary isolated dendritic cells (Bechetoille et al., 2011; Chau et al., 2013; Saalbach et al., 2015), and human epidermis-derived macrophages (Bechetoille et al., 2011). The incorporation of neurons and adipocytes also provide an additional aspect of immune and disease modeling in 3D skin equivalents. Vidal et al. produced a full thickness 3D skin construct containing human induced neuronal stem cells within an adipose scaffold hypodermis layer to model an immune-competent skin equivalent (Vidal et al., 2018). Moreover, the hydrogel composition may influence immune responses *in vitro*. Lohmann et al. demonstrated that hydrogels composed of star-shaped PEG and heparin (a type of GAG) are able to bind chemokines by electrostatic interactions. This hydrogel worked as an effective chemokine scavenger and reduced inflammatory processes in an *in vivo* model of delayed wound healing in mice (Lohmann et al., 2017). Likewise, an immune-competent hydrogel comprised of both a hypodermis containing lipoaspirate (adipocytes, pre-adipocytes, endothelial cells, smooth muscle cells, and macrophages) and human induced neural stem cells was fabricated, from which time-dependent inflammatory responses were observed (Vidal et al., 2018).

### Outlook of skin disease models in 3D

It is important to consider the benefit of 3D skin constructs when modeling skin diseases *in vitro*. For example, the incorporation of relevant cell types that influence wound healing *in vivo* such as Langerhans cells, dermal macrophages, neutrophils, and adipocytes are necessary. Hence, the classic *in vitro* skin models cannot be used to model physiological wounds. Moreover, assessment of drugs or wound dressings in the current *in vitro* models is inadequate or impractical.

With respect to the incorporation of cell types into 3D skin models, several commercially available skin models have been developed that include melanocytes (e.g. MelanoDerm™, MatTek corp.; epiCS®-M, ATERA SAS & CellSystems Gmbh; and SkinEthic ™ RHPE, EPISKIN, subsidiary of L'Oreal) for the testing of sun care products. Biedermann et al. demonstrated that melanocytes seeded in a 3D skin model retain their phenotype (expression of BCL2, SOX9, and MITF) for over 15 weeks after transplantation onto immune-incompetent rats, which has important clinical implications for matching transplanted skin with patient skin color (Biedermann et al., 2015). Additionally, the presence of melanocytes is crucial for the investigation of vitiligo that has yet to be modeled *in vitro*.

Nevertheless, the infiltration of leukocytes, such as non-resident macrophages and granulocytes, from vasculature into the hydrogel has yet to be established. This would require development of a perfusion system that includes vasculature and lymphatic networks as was aforementioned regarding physiological nutrient and waste transport. What is more, disease models should include the incorporation of hair, glands, and tactile corpuscles. To date, these macro-structures/appendages are absent in 3D skin equivalents but are significant for developing models of *in vitro* diseases that are currently only modeled in animals, such as alopecia and acne vulgarus. In this regard, bioprinting technology holds great promise in the development of diseased skin models. In addition, the incorporation of peptides that illicit immune responses or promote matrix degradation mimicking disease may be possible with electrospinning techniques. First and foremost, the challenge remains for the technology to produce improved healthy skin models with all of these components with the production efficiency of the standard casting technology.

## The future of 3D skin fabrication *in vitro*

Current technologies with respect to 3D tissue fabrication facilitate the automated production of reproducible and functional skin tissue. For instance, biofabrication techniques already enable the production of large-scale pigmented skin transplants in the range of 100 cm^2^ (Min et al., 2018). In addition, commercially available constructs for *in vivo* applications exist such as Apligraf® (Eaglstein and Falanga, 1998), Theraskin®, Dermagraft (Hart et al., 2012), and OrCel® and *in vitro* applications such as EpiSkin (EPISKIN SA a subsidiary of L'Oreal) (Roguet et al., 1994), Epiderm™ (MatTek Corp.) (Cannon et al., 1994), Leiden epidermal skin model and Fully Human Skin Model (Biomimiq a division of Aeon Astron Europe B.V.), EpiCS® RHE (CellSystems® GmbH and ATERA SAS) and LabCyte EPI-MODEL (Japan Tissue Engineerging Co., Ltd.). With that being said, it is evident that collaborations between industry and academia can further progress the development of these models toward a more physiologically relevant construct and encourage their use in basic science to replace 2D tissue culture.

Moreover, challenges in the production and use of 3D skin models for basic science lies in affordability. Both bioprinting and electrospinning not only enable the ease of production of such constructs but also contribute to low-cost strategies for development of 3D skin tissues. Bioprinting and electrospinning have already been used in combination instruments, though the addition of new technologies for use in tandem may aid in the production of vasculature, appendages, and constructs easily modulated for disease modeling (Figure 2).

### Toward a more relevant *in vitro* 3D skin model

Although standard 3D skin models are a major improvement when compared to 2D culture, their minimalism still does not yet reflect the *in vivo* situation. Key areas necessary to produce a more relevant 3D skin model include: (1) the creation of a well-defined physiological matrix and microenvironment, (2) inclusion of additional specialized cell types, and (3) ease of production with new fabrication techniques.

**Producing well-defined physiological matrix components** goes further than collagen and fibrin. These proteins and fibers are highly modified by post-translational modifications each of which possesses specific functionalities. Therefore, although combinations of commercially available collagen and other natural or synthetic polymers appears to be the strategy of many researchers, alternative methodology for the production of physiological matrices should be considered. Perhaps, similarly to CRISPR technologies, a biochemical mechanism can be exploited to fabricate physiological matrix with appropriate post-translational modifications that is also compatible with bioprinting technologies. Such an angle might be the use of isolated endoplasmic reticulum programmed to produce collagens and elastins that is bioprinted within a scaffold support.

The formation of physiologically relevant microenvironments adds additional complexity as it is difficult to measure such parameters *in vivo*. Additional research to identify the ideal culture conditions for sebocytes, isolated glands, and hair follicles can give us inklings into the microenvironments necessary to recapitulate within a 3D printed skin construct. Meanwhile, the integration of vasculature-like structures lends to the optimization of physiological nutrient and oxygen delivery as well as waste product removal. These are already rapidly advancing due to electrospinning and bioprinting, though with the addition of new novel techniques that can be combined with bioprinting, the ease of production can improve.

As mentioned briefly above, the **addition of specialized cell types** (e.g. sebocytes, Merkel cells etc.) may promote the sustained culture of 3D skin constructs *in vitro*. Addition of inflammatory cells goes beyond the need to recapitulate disease. Specifically resident tissue macrophages are responsible for the clearing of terminated cells that may otherwise result in a decline in overall tissue health if left unresolved. Therefore, these cells are required when producing healthy and physiologically relevant skin constructs to sustain the overall health of the tissue during culturing. 3D printing can facilitate in the placement and inclusion of such cell types into 3D skin constructs at present, thus sustainable tissue culture of 3D skin constructs is in the immediate future. Furthermore, the spatial positioning provided by biofabrication techniques can enable the production of appendages and macrostructures within skin tissue that include these specialized cell types for the formation of glands and hair follicles.

Each aspect of physiological skin is possible with current **biofabrication techniques** or the development of novel techniques with a specific purpose and compatibility with current technologies (Figure 2). For one, the combination of MESW and 3D bioprinting is becoming more commonplace and the first commercially available bioprinters are on the market. The integration of additional electrospinning techniques such as cryo-electrospinning, salt leaching electrospinning, cold-plate electrospinning, emulsion electrospinning or air flow electrospinning may also be promising for the production of specific structural components. Moreover, the development of novel printheads for multipurpose bioprinting is optimal. Novel technologies being developed at present should consider the ability to be integrated for use with other technologies and not as a stand-alone product. This will aid in the ease of tissue production and cost-effectiveness of such tissues (Figure 2).

### Future perspective

The development and evolution of *in vitro* 3D skin tissue over the past 70 years provides skin models that can replace the need for animal experimentation in pre-clinical testing and advances the capabilities for personalized medicine. Unfortunately, with respect to production of 3D skin, we still face many of the same issues that troubled scientists in the middle of the twentieth century, including physiological oxygen and nutrient delivery with a perfused vasculature, complex structures such as glands and tactile corpuscles, as well as readily accessible and reproducible constructs for use in research laboratories. Furthermore, sustained tissue culture and skin disease modeling will become a reality once immune-competent cells are successfully integrated, both into hydrogels as well as within a circulating vasculature. The convergence of different biofabrication techniques, such as bioprinting and electrospinning will address not only the incorporation of immune cells into the skin model, but also appendages to increase the ability for one to create a physiological skin for personalized medicine and other pre-clinical applications (e.g. drug testing) (Figure 2). The production of necessary physiologically relevant skin components from ECM to microbiome are feasible with current bioengineering technologies, though additional advancements to existing technologies and development of completely novel technologies will provide cost-effective and reproducible generation of physiological skin *in vitro*.

## Author contributions

MJR and KW-K conceptualized the manuscript. MJR, KW-K, AJ, and MR wrote the manuscript. MJR designed and illustrated the figures.

### Conflict of interest statement

The authors declare that the research was conducted in the absence of any commercial or financial relationships that could be construed as a potential conflict of interest.
